# Gait speed in clinical and daily living assessments in Parkinson’s disease patients: performance versus capacity

**DOI:** 10.1038/s41531-021-00171-0

**Published:** 2021-03-05

**Authors:** Arash Atrsaei, Marta Francisca Corrà, Farzin Dadashi, Nuno Vila-Chã, Luis Maia, Benoit Mariani, Walter Maetzler, Kamiar Aminian

**Affiliations:** 1 Laboratory of Movement Analysis and Measurement, Ecole Polytechnique Fédérale de Lausanne (EPFL), Lausanne, Switzerland; 2 Gait Up S.A., Lausanne, Switzerland; 3 Instituto de Ciências Biomédicas Abel Salazar (ICBAS), Porto, Portugal; 4 Centro Hospitalar Universitário do Porto (CHUP), Porto, Portugal; 5 Klinik für Neurologie, Universitätsklinikum Schleswig-Holstein (UKSH), Kiel, Germany

**Keywords:** Parkinson's disease, Biological techniques

## Abstract

Gait speed often referred as the sixth vital sign is the most powerful biomarker of mobility. While a clinical setting allows the estimation of gait speed under controlled conditions that present functional capacity, gait speed in real-life conditions provides the actual performance of the patient. The goal of this study was to investigate objectively under what conditions during daily activities, patients perform as well as or better than in the clinic. To this end, we recruited 27 Parkinson’s disease (PD) patients and measured their gait speed by inertial measurement units through several walking tests in the clinic as well as their daily activities at home. By fitting a bimodal Gaussian model to their gait speed distribution, we found that on average, patients had similar modes in the clinic and during daily activities. Furthermore, we observed that the number of medication doses taken throughout the day had a moderate correlation with the difference between clinic and home. Performing a cycle-by-cycle analysis on gait speed during the home assessment, overall only about 3% of the strides had equal or greater gait speeds than the patients’ capacity in the clinic. These strides were during long walking bouts (>1 min) and happened before noon, around 26 min after medication intake, reaching their maximum occurrence probability 3 h after Levodopa intake. These results open the possibility of better control of medication intake in PD by considering both functional capacity and continuous monitoring of gait speed during real-life conditions.

## Introduction

Motor impairments in Parkinson’s disease (PD) are often characterized by tremor, postural instability, and reduced gait speed^1,2^. While the cause of PD is unknown, degeneration of dopaminergic nerve cells is associated with reduced motor function and impaired movement control. Therefore, PD treatments focus on the control of motor and non-motor symptoms using dopamine compensation, mainly with Levodopa, and surgical methods such as deep brain stimulation^3^.

To monitor the progression of disease and symptoms, assessment scales such as the Unified Parkinson’s disease Rating Scale (UPDRS) are being used widely by clinicians. Although these scales have been shown to have reliable clinimetric characteristics^4^, they cannot be obtained continuously and are dependent on the rater^5,6^. More objective assessments can include timed tests in the lab in which gait speed can be calculated by measuring the time taken to traverse a predefined distance by stop-watch, e.g., 20-m walk test.

With inertial measurement units (IMUs), gait parameters can be obtained accurately providing objective outcome measures^7–11^. Based on the IMU signals or derived gait parameters, one can classify early PD^12^, investigate subtle differences among PD patients^13^, predict freezing of gait^14,15^, monitor PD symptoms^5^, and the Levodopa response^16,17^ in long-term daily activities. Among various gait parameters, gait speed is often considered as the sixth vital sign^18^ and has been shown to be a reliable measure in diagnosis^19^ and a marker of functional decline^20,21^. As this parameter contains both spatial, i.e., stride length, and temporal, i.e., gait cycle time, aspects of gait, it has a strong discriminative power among patient populations^12^.

Being wearable, IMUs allow gait to be assessed in both clinical and domestic environments. However, as the International Classification of Functioning Disability and Health (ICF) model suggests, there is a difference between the assessments performed in the clinic which reflects functional capacity and the assessments performed during daily activities, which are more indicative of the actual performance of the individuals^22^. For instance, it has been shown that during daily activities, gait speed can decrease by 30% compared to the clinic in PD patients^23^. A basic explanation for this different behaviour is that mobility is not only affected by the sensorimotor system but also by psychological factors^24–27^. Patients are more focused on the task and try to achieve better results in the presence of a clinician than during their actual performance in everyday life^5^. Moreover, the context of the environment is different at home or outdoor where there are multiple obstacles and more complexity compared to the clinical setting^27,28^. Therefore, unsupervised assessments at home can provide additional information through long-term monitoring^29^. Furthermore, it would also be possible to capture rare incidents such as falls or stage before an injury which may not be measurable during a clinical visit.

Hence, domestic and clinical assessments can be considered as associated but separate domains of physical function^30^. Recent studies have been trying to discover the associations between clinical and home assessments. In a group of PD patients, gait and postural transition parameters were evaluated at the clinic and home^23^. It was observed that no significant correlation between clinical and home measurements exists for the patients, even for the same parameter. This study was limited in a sense as for the assessments performed at home, the wide distribution of parameters such as gait speed was condensed to an average value. As a consequence, the large variety of gait speed at home was neglected.

It has been shown that the extreme values of gait or balance parameters of home-based monitoring are more closely associated with the laboratory-based measurements^31–33^. In a study, it was observed that the differences between PD patients and healthy older adults become more evident during daily living conditions because of the reduced attentional input in a real-life setting^34^. However, for some parameters such as gait speed, it has been shown that during free-living conditions, only longer walking bouts could distinguish the two populations. The turning parameters have been also studied in PD patients with and without risk of falls^35^. The results of this study suggested that fear of falls affects the turning behaviour of the patients differently in the clinic and at home. The association of the laboratory and home-based measurements with conventional clinical assessments, e.g., the UPDRS, has been also studied. In a large group of PD patients, the authors showed that 46% of the UPDRS variance was explained by the demographic data, clinical and home assessments. From this portion, most of the variance (62%) was explained by daily living measurements^36^.

These studies have revealed that there is a difference between the clinical and home assessments even for the same parameter^27^. The previous studies are mostly based on correlation analysis that showed the association and the difference between clinical and home measurements. Yet, the relationship between these two assessments is not fully understood. Previous studies have not shown under what conditions these differences between clinic and home are minor. Knowing these conditions, clinicians can have a better estimate of how much extent patients’ capacity is being used in real-life.

Therefore, in this study, we aimed towards investigating the conditions in which the clinical and home assessments become closer. More specifically, we focused on the gait speed and we have answered the following two research questions:

1. Do patients with PD have the same preferred gait speed at the clinic and home?
2. Under what condition does the PD patient performance measured by gait speed in free-living conditions reach the capacity measured by gait speed in the clinic?

The novelty of this study is the way we quantified gait speed distribution particularly, during daily activities in PD patients. In previous studies, the distribution has been mostly condensed to one mean and standard deviation values limiting the information we can get from this wide distribution. In this study, by including several walking tests in the clinic rather than a single gait test, we investigated the hypothesis of a bimodal gait speed distribution during both clinical and home assessments. Moreover, we have shown that how the medication state, the time of the day, and the duration of walking bouts can contribute to the difference between capacity and performance. This information can provide a better understanding of the relationship between medication intake and the resulting increase in performance at home compared to the patients’ capacity.

## Results

### Distribution of gait speed at the clinic and home

**Fig. 1:**
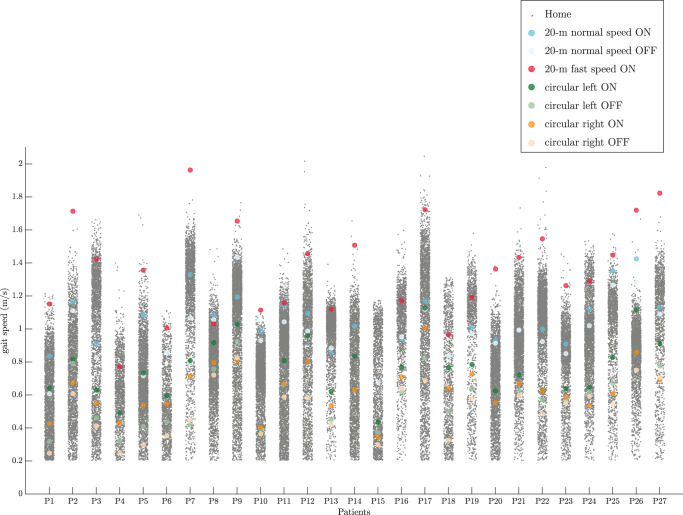
Distribution of gait speed at home and the average values of the gait speed for the clinical assessments for each patient. For each patient, the average gait speed during the 20-m walking test was considered as their capacity.

The mean gait speed during clinical assessments was compared to the distribution of the gait speed at home for all the patients (Fig. 1). The average value of the 20-m walk test with fast speed, considered as the capacity of the patients, were near to or even higher than the maximum values of the gait speed measured at home. Furthermore, the average value of the circular walking tests was lower than the other clinical assessments. The average duration of the straight walking tests for all the patients was 18.5 ± 3.8 s.

**Fig. 2:**
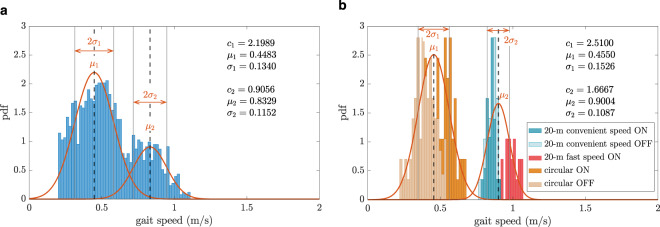
An example of the gait speed probability density function (pdf) for one of the patients (P6). The distribution is shown at (**a**) home and (**b**) at the clinic. The red fitted curves are the first and second terms of the bimodal Gaussian distribution introduced by Eq. 1, the parameters $c_1$, $c_2$, $\mu _1$, $\mu _2$, $\sigma _1$, and $\sigma _2$ are the Gaussian mixture model parameters defined in Eq. 1.

For a typical patient, the histogram of the gait speed as the probability density function distribution is shown in Fig. 2 along with the fitted Gaussian mixture models during daily activities and all the clinical assessments. The bimodal distribution of the gait speed at both home and clinic can be inferred from this figure. The patient had two preferred gait speeds, a lower ($\mu _1$) and a higher one ($\mu _2$) during both clinical and home assessments. The standard deviations from these two preferred speeds were denoted by $\sigma _1$ and $\sigma _2$. For this specific patient, the preferred gait speeds at home (0.44 and 0.83 m/s) were close to the preferred speeds at the clinic (0.45 and 0.90 m/s).

For the clinical measurements, the distribution was also shown coloured with the type of the test. The circular walking tests constructed the left part of the distribution and the straight walking tests constructed the right part of the distribution.

**Fig. 3:**
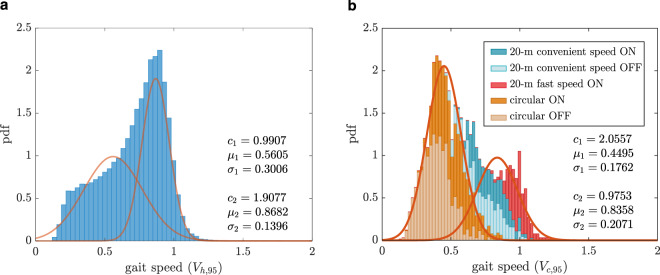
The gait speed probability density function (pdf) for all the patients together. The distribution is shown at (**a**) home normalized by $V_{h,95}$ and (**b**) the clinic normalized by $V_{c,95}$, the red fitted curves are the first and second terms of the bimodal Gaussian distribution introduced by Eq. 1.

To evaluate the existence of bimodal Gaussian distribution in the whole group of the patients, for each patient their gait speed distributions in the clinic and at home were normalized by the 95th percentile of the respective distribution ($V_{c,95}$ for clinical assessment, $V_{h,95}$ for home assessment). The gait speed distributions in both clinic and daily activity are depicted by considering all patients congregated (Fig. 3). During the clinical assessment, the circular walking tests lay more on the left of the distribution, the straight walking tests with convenient speed were in the middle and the fast walking tests were at the right of the distribution.

The fitting quality of the bimodal Gaussian distribution estimated by Ashman’s D value was higher than 2 for all the clinical assessments. However, for three patients (P5, P8, and P26), this value was below 2 during their home assessment, meaning that there was not a clear separation between the modes of gait speed distribution at home. For all the remaining patients, the means ($\mu _1$ and $\mu _2$) and standard deviations ($\sigma _1$ and $\sigma _2$) were compared between the clinic and home with the Wilcoxon rank-sum test (Table 1).

**Table 1:** Comparison of the preferred gait speeds along with their corresponding deviations between clinic and home, the significance level (* in bold) was set to 0.05, two-sided Wilcoxon rank sum test, Cohen’s d was calculated for effect size.

	Home (m/s)		Clinic (m/s)		Comparison		Correlation	
	Median	IQR	Median	IQR	*p*-value	Cohen’s d	*ρ*	*p*-value
$\mu _1$	0.47	[0.44, 0.73]	0.63	[0.47, 0.71]	0.3173	0.29	**0.52**	0.0084*
$\mu _2$	1.00	[0.88, 1.14]	1.02	[0.90, 1.41]	0.5028	0.39	**0.61**	0.0015*
$\sigma _1$	0.17	[0.13, 0.26]	0.08	[0.07, 0.15]	<0.001*	−1.07	0.06	0.7897
$\sigma _2$	0.14	[0.11, 0.16]	0.14	[0.08, 0.23]	0.6725	0.25	−0.02	0.8898

No significant difference was observed between the means ($\mu _1$ and $\mu _2$) and the standard deviation corresponding to the higher preferred gait speed ($\sigma _2$) between the clinical and home assessments. However, the standard deviation corresponding to the lower preferred gait speed ($\sigma _1$) was significantly higher at home compared to the clinic (*p*-value < 0.001). These results show that the patients had on average the same preferred gait speeds at the clinic and at home with the same deviation from the higher preferred gait speed. However, their gait speed variation around the lower preferred gait speed was significantly higher during daily activities. A moderate correlation was found for the higher preferred gait speed ($\mu _2$) between the clinic and home ($\rho = 0.61$, *p*-value = 0.0015, 95% confidence interval: 0.28: 0.81). The correlation between the lower preferred gait speed ($\mu _1$) was also moderate ($\rho = 0.52$, *p*-value = 0.0084, 95% confidence interval: 0.15: 0.77). No significant correlation was found for the standard deviations ($\sigma _1$: $\rho = 0.06$, *p*-value = 0.7897, 95% confidence interval: −0.34: 0.46 and $\sigma _2$: $\rho = - 0.02$, *p*-value = 0.8898, 95% confidence interval −0.32: 0.48).

**Fig. 4:**
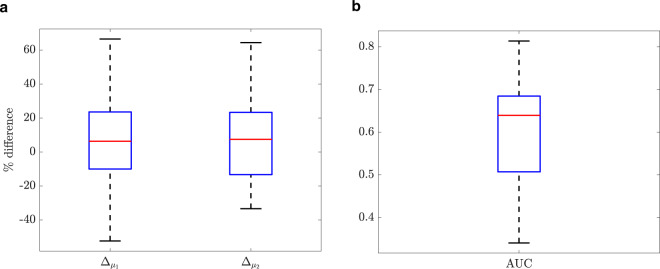
The boxplots comparing the gait speed distribution between the clinic and home for all the patients. (**a**) The percentage of the difference between clinic and home for preferred gait speeds $\mu _1$and $\mu _2$, (**b**) the area under the ROC curve (AUC) of ${\mathrm{CDF}}_{\mathrm{clinic}}$ versus ${\mathrm{CDF}}_{\mathrm{home}}$: Center line: median; box limits: upper and lower quartiles; whiskers: 1.5 × interquartile range.

${{\Delta }}_{\mu _1}$and ${{\Delta }}_{\mu _2}$ as the percentage of the differences for preferred gait speeds between clinic and home were shown in Fig. 4a. The median values are 6% and 7%, for ${{\Delta }}_{\mu _1}$and ${{\Delta }}_{\mu _2}$, respectively. The 25th and 75th percentiles are <23%, and the upper and lower adjacent values can reach up to 60%.

The AUC values that present the similarity of the cumulative distribution functions of clinic and home were shown in Fig. 4b. The median value was obtained as 0.64 and the 25th and 75th percentiles as 0.51 and 0.68, respectively.

**Fig. 5:**
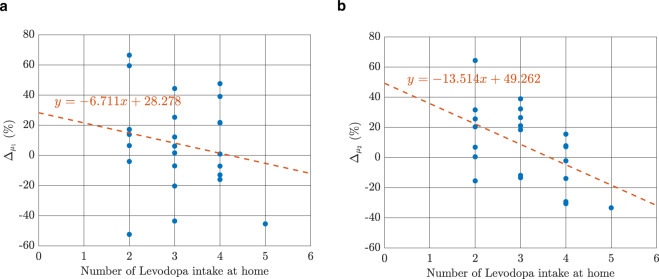
The relationship between the number of medication doses taken during the interval of data recording in home assessment. This linear relationship is shown for (**a**) $\Delta _{\mu _1}$and (**b**) $\Delta _{\mu _2}$.

The correlation between the number of medication doses taken during the course of data recording and ${{\Delta }}_{\mu _1}$ was $\rho = - 0.19$ (*p*-value = 0.3649, 95% confidence interval: −0.55: 0.23). The correlation between number of medication intakes and ${{\Delta }}_{\mu _2}$ was $\rho = - 0.50$ (*p*-value = 0.0126, 95% confidence interval: −0.75: −0.12). Plotting the number of medication doses intake versus ${{\Delta }}_{\mu _1}$ and ${{\Delta }}_{\mu _2}$ in Fig. 5 revealed that patients with a higher number of Levodopa intakes during daily activities performed faster at home (${{\Delta }}_{\mu _2} < 0$) while patients with a lower number of Levodopa intakes performed faster in the clinic (${{\Delta }}_{\mu _2} > 0$).

### Exceptional Strides

**Fig. 6:**
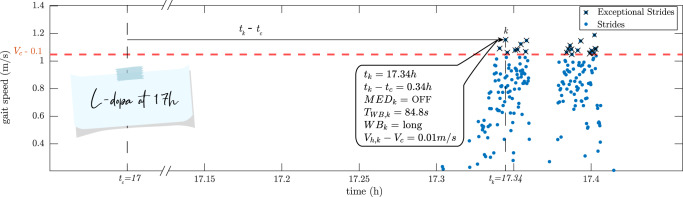
The information extracted for Exceptional Stride *k* for one of the patients as an example. Each blue dot shows the gait speed of a gait cycle at a specific time of the day during daily activities. This patient took Levodopa at time $t_c = {17\mathrm{h}}$. $V_c$ is the capacity of the patient, i.e., gait speed during fast walking test in the clinic. The Exceptional Strides have marked with black crosses. *k* is one example of the Exceptional Strides with the information extracted according to section II-F. No walking with a duration of more than 15 s occurred after 17 h and before 17.3 h.

Regarding the Exceptional Strides, the information concerning one Exceptional Stride *k* (section II-F) as an example, is shown for one of the patients (Fig. 6). This specific Exceptional Stride happened 0.34 h (20.4 min) after the last Levodopa intake at 17:00. Therefore, it happened during the predefined OFF state. Furthermore, this stride belonged to a walking bout with a length of 84.8 s considered as a long walking bout. The gait speed of this stride was 0.01 m/s higher than the patient’s capacity ($V_c$).

Out of 27 patients, 3 patients did not have any Exceptional Stride in their home assessment (P2, P7, and P27). Furthermore, for one of the patients (P15), no data was present from their 20-m straight walk test with fast speed as depicted in Fig. 1. Stacking the data from the remaining 23 patients together, the aforementioned parameters were given in Table 2.

**Table 2:** The parameters of Exceptional Strides for all the patients except P2, P7, P15, and P27.

	Median	IQR
Number of Exceptional Strides	104	[32, 557]
Normalized number of Exceptional Strides (%)	3.4	[0.9, 25.1]
*t*_*k*_ (h)	11.7	[10.6, 14.6]
*t*_*k*_−*t*_*c*_ (h)	2.80	[2.03, 3.42]
MED_*k*_ = ON (%)	27.4	[3.5, 75.8]
MED_*k*_ = OFF (%)	3.9	[0.2, 26.1]
*T*_WB*,k*_ (s)	46.2	[26.1, 129.4]
WB_*k*_ = short (%)	0.9	[0, 10.3]
WB_*k*_ = medium (%)	7.0	[3.9, 19.4]
WB_*k*_ = long (%)	89.5	[72.8, 95.1]
*V*_*h,k*_−*V*_*c*_ (m/s)	−0.02	[−0.06, 0.04]

It can be observed that a median of 104 Exceptional Strides existed from all the 23 patients (see Table 2). For each patient, the number of their Exceptional Strides was normalized by their total number of strides. It can be seen that 3.4% of their gait cycles had a speed higher than or equal to their capacity at the clinic.

A negative but insignificant trend was observed between the amount of Exceptional Strides and UPDRS-III ($\rho = - 0.10$ *p*-value = 0.6344, 95% confidence interval: −0.47: 0.30). Moreover, a positive but insignificant relationship was found between the amount of Exceptional Strides and number of Levodopa intakes ($\rho = 0.17$ *p*-value = 0.4144, 95% confidence interval: −0.23: 0.52).

**Fig. 7:**
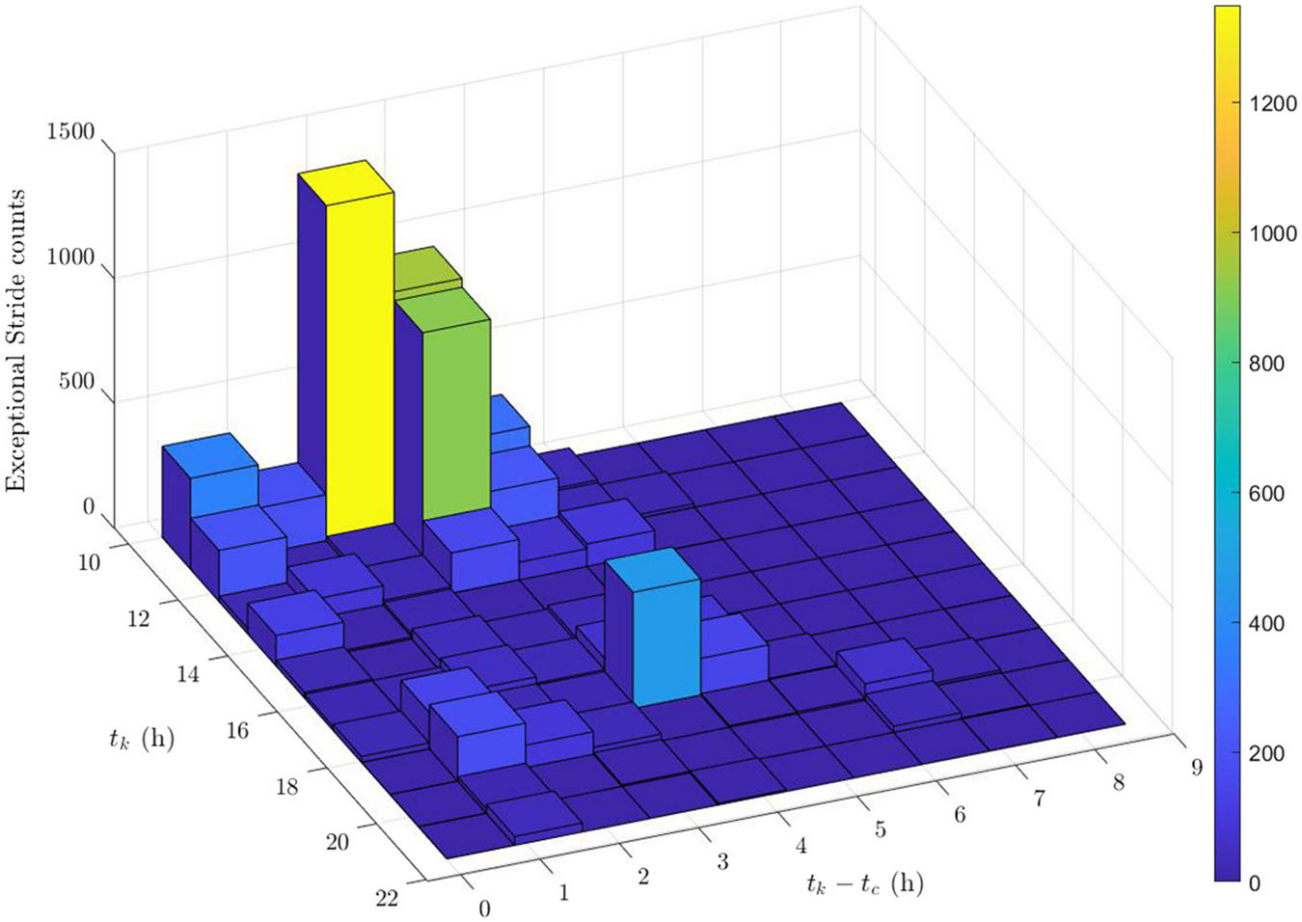
3D Histogram plot of Exceptional Stride time of occurrence ($t_k$) and their time difference from their corresponding previous medication intake ($t_k - t_c$). The yellow bar demonstrates the highest peak of the Exceptional Strides that occurred around 10:00 to 10:30 and had a time difference of ~2 h with their previous medication intake. Therefore, they correspond to the medication doses taken around 8:00 to 8:30.

Exceptional Strides occurred at a median of 11.74 h or a bit before noon (11:44). The 3D histogram plot for the time of occurrence of the Exceptional Strides (*t*_*k*_) as well as their time difference with regard to their previous medication intake ($t_k - t_c$) is shown in Fig. 7. In this figure, the yellow bar demonstrates the highest peak of the Exceptional Strides that occurred around 10:00 to 10:30 and had a time difference of ~2 h with their previous medication intake. Therefore, they correspond to the medication doses taken around 8:00 to 8:30. Other peaks can be observed around 12:00 and 17:30.

**Fig. 8:**
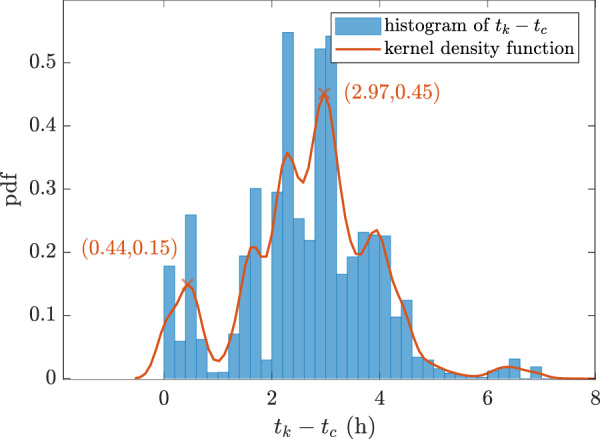
The probability distribution function (pdf) of Exceptional Strides in relation to medication intake time (blue) with the fitted Gaussian mixture model (red). Two peaks can be distinguished from the kernel smoothening function at 0.44 and 2.97 h.

Regarding the time difference between the Exceptional Strides and their corresponding last medication intake, the median value was 2.80 h which states that most of the Exceptional Strides happened 2.80 h after taking Levodopa. The probability distribution function (pdf) of the time differences were plotted in Fig. 8 along with the fitted kernel density smoothening function. Two peaks can be distinguished from the kernel smoothening function at 0.44 and 2.97 h. This implies that around half an hour and three hours after taking the medication, there is a high probability of having a gait speed equal or greater than the capacity at the clinic. Moreover, a sharp drop can be observed at ~1 h after taking the medication.

Furthermore, the probability of having an Exceptional Stride during ON state was higher than during OFF state (Table 2).

While the median of the walking bout duration in which the Exceptional Strides had occurred ($T_{WB,k}$) was 46.17 s, most of the Exceptional Strides happened in long walking bouts, i.e., walking bouts with a duration of more than 60 s. 89.5% of the Exceptional Strides belonged to long walking bouts while this amount was reduced to 7.0% and 0.9% in medium and short walking bouts, respectively.

Finally, the median difference between the gait speed of the Exceptional Strides and the capacity ($V_{h,k} - V_c$) was obtained as −0.02 m/s (Table 2).

## Discussion

In this paper, we aimed to investigate under what conditions the clinical and home measurements demonstrate a close association. In previous studies, it had been proven that there are differences even for the same parameter obtained during clinical and home assessments^23,31,34,37–40^. However, to the best of our knowledge, it has not been investigated under what circumstances the gap between clinical measurements and real-life daily activities becomes smaller.

Gait speed was extracted during functional tests performed at the clinic and during daily activities in real-life settings. Several walking tests were performed at the clinic during both ON and OFF states to capture different aspects of the patients’ gait. During daily activities, we discarded the walking bouts with a duration of <15 s to include walking bouts with a steady-state gait speed. This value is reasonable as the duration of the straight walking tests during the clinical assessment was around 18 s making the comparison between clinic and home fairer. It was shown that the 20-m straight walking test with fast speed lay at the extreme end of the gait speed distribution at home (Fig. 1). This is in line with what has been previously reported in the literature^27,31,41^. Comparing the gait speed obtained during daily activities and a 4-m walk test at the clinic in community-dwelling participants, one previous study showed that the high percentiles of the gait speed distribution at home had higher correlations with the 4-m walk test at the clinic^31^.

Specifically, for three patients, i.e., participants #16, 18, and 19, their fast walking test at the clinic had relatively slower speed compared to their maximal performance at home as there were many gait cycles with a higher speed at home (Fig. 1). While due to psychological factors people behave differently in different settings^28^, we believe that some other reasons can also explain this difference. We checked the assessment data of these patients in more detail, and found that they all performed their walking tests in the clinic formally during best ON medication, i.e., about 90 min after their last Levodopa intake. Therefore, we were reassured that the protocol of the test regarding the assessment time after the medication intake was respected for these patients. Moreover, their treatment response as defined by the UPDRS-III scores (participant #16: 26 points during OFF, 20 points during ON; participant #18: 31 versus 12; participant #19: 22 versus 8) indicates good Levodopa response. Nevertheless, we believe that the effect of the medication can be different for each patient and patients can respond differently to dopaminergic medication especially concerning pharmacodynamic aspects. This, in fact, shows that home assessment can have complementary information to clinical assessment and may give us a better insight about the actual capacity of the patients. While the reasons for these differences in the clinic versus home behaviour remain unclear, our study may stimulate further investigation in this area of research.

The gait speed distribution during both of the clinical assessments and daily activities followed a bimodal distribution for almost all the patients. This indicates that patients had two different preferred gait speeds. During clinical assessment, this phenomenon is because patients were assessed basically under two groups of walking tests, demanding as well as simpler ones. During home assessment, we can assume that the lower preferred gait speed is more attributed to shorter walking bouts that occur more indoors and higher preferred gait speed to the longer walking bouts that might occur more outdoors. Although we did not ask the patients to register the information about their indoor or outdoor activities, having this information could have confirmed our hypothesis. This bimodal phenomenon has been shown in previous studies for gait speed^31^ and cadence^42^ in community-dwelling adults during daily activities. In this study, we have confirmed this phenomenon in PD patients during daily living measurements. The advantage of such quantification of gait speed distribution is to preserve the information of this wide distribution rather than condensing it to one mean and standard deviation value.

In Fig. 2 and 3, it was shown that the circular walking tests composed the lower scales of the gait speed distribution while the straight walk tests constructed the higher gait speeds. This is not surprising as patients can have a lower gait speed in more demanding tasks. In a study on older fallers, it was shown that the gait speed obtained during dual-task walking tests corresponded better to the daily activities as opposed to the usual walking test^41^. This shows that performing more demanding walking tests in the clinic can give a better view of the patients’ performance at home and clinicians can adapt or choose the most relevant clinical assessments. In other words, more demanding walking tests such as circular walk tests or dual-task tests represent the patients’ lower preferred gait speed and simple walking tests such as straight walk tests represent the patients’ higher preferred gait speed during daily activities.

Comparison of the bimodal distribution between clinic and home showed that patients had on average the same preferred gait speeds in both of the settings (Table 1). There was a significant difference between the two settings for the variations from the lower preferred gait speed ($\sigma _1$) but not from the higher gait speed ($\sigma _2$). Patients had higher variability for their lower preferred gait speed at home compared to the clinic. This can be explained by the complex context of the environment in real-life settings, e.g., turns, curved paths, obstacles, which causes people to continuously adapt their gait speed^37^. However, the variations around the higher preferred gait speed ($\sigma _2$) was not significantly different between real-life and clinical setting. This might be because the higher preferred gait speed expresses the capacity of the patients which might stay constant between clinic and home. This can also explain the higher correlation for the higher preferred gait speed ($\rho = 0.61$) between lab and home compared to the lower preferred gait speed ($\rho = 0.52$). Another contributing factor can be the use of different vestibular systems when we walk slowly or fast^43,44^.

While the statistical test did not show a significant difference between clinic and home for the preferred gait speeds ($\mu _1$ and $\mu _2$), this lack of significance can be due to lack of power. To this end, we introduced additional parameters (${{\Delta }}_{\mu _1}$ and ${{\Delta }}_{\mu _2}$) to look at the difference between clinic and home more deeply. ${{\Delta }}_{\mu _1}$ and ${{\Delta }}_{\mu _2}$ showed that for most of the patients, the difference between clinic and the home was <23% while there were few patients that had a larger difference of up to around 60% between clinic and home (Fig. 4a). The AUC values that were on average about 0.64 confirmed that the cumulative distribution function of gait speed in clinic and home are comparable (Fig. 4b).

The reason for this difference between clinic and the home was partly explained by the variation in PD as there was a significant and moderate correlation between the number of Levodopa intakes throughout the day and ${{\Delta }}_{\mu _2}$ (Fig. 5). These results suggest that higher numbers of daily Levodopa intakes have a positive impact on the preferred walking speed at home, especially in the “capacity area” ($\mu _2$). However, we should also consider that patients with a lower number of Levodopa intakes tend to respond better during clinical assessments. Another similar but independent reasoning can be the rationale behind why certain PD patients may get a little number of daily Levodopa prescribed, e.g., because they may not be able to manage a complex medication regimen. Considering our limited sample size, whatever the reasons are for this observation, such analyses can serve as the first steps into a better understanding of the relation between medication intakes and the difference between clinical and home assessments.

Therefore, to answer our first research question which was whether patients have the same preferred gait speed in the clinic and at home, we showed that by performing gait assessments under different conditions in the clinic, we can cover a wide range of gait speeds that can reach on average similar bimodal distribution observed in real-life. Nevertheless, daily-life measures can still provide complementary information to the clinical assessments^27,30^.

To answer the second research question which was investigating the instances in which the patients’ performance reaches their capacity, we introduced and detected the Exceptional Strides for each patient during real-life conditions. These strides express the ability of the patient to reach equal or greater gait speed than the fast speed in the clinic ($V_c$) considered as the capacity of the patients. We considered a threshold of 0.1 m/s to compensate for the measurement errors. This value can be justified by the error of the employed algorithm (around 5 cm/s) to extract gait speed as shown in^45^. Exceptional Strides constituted only 3.4% of the total strides of the patients (Table 2). This reveals that in very small part of daily activities patients went beyond their capacity.

Although not significant, a negative relation was found between UPDRS-III and the amount of Exceptional Strides meaning that patients with higher UPDRS-III can have a lower number of Exceptional Strides. Moreover, the positive but insignificant relation between the amount of Exceptional Strides and the number of Levodopa intakes taken during the day suggests that patients with higher amounts of Exceptional Strides might have taken a higher number of medication doses. However, more evidence with a larger dataset is needed to confirm these findings.

Histogram plot of Exceptional Strides time of occurrence (Fig. 7) showed that most of the Exceptional Strides happened before noon. This confirms the finding in the literature that PD patients with early or moderate stage of the disease have similar pattern of diurnal activity and are more active in the morning with a late morning peak^46^. This may be explained by being more active and having more walking bouts that occurred in the morning. These strides decreased in the afternoon reaching a minimum at 13:30 which might be due to a decrease in activity levels after lunch. Exceptional Strides increased again reaching their maximum around 17:00 in the evening which can again be due to the recovered energy before the end of the evening. Moreover, some of the patients were going to work; therefore, coming back from work can be another potential explanation to have Exceptional Strides at 17:00. However, the Exceptional Strides count was still approximately only one third compared to the morning. Having the Exceptional Strides mostly in the morning can also be due to the study design as the patients had to go back home from the hospital, therefore they might have had more long walking bouts and consequently more Exceptional Strides in the morning.

The effect of Levodopa might be considered as maximum, ~3 h after taking the medication as the Exceptional Strides occurred mostly at this time (Table 2). This is in line with a previous study that presented a model for Levodopa medication effect in finger tapping tests^47^. It was shown that the tapping frequency increased around 30 min after taking Levodopa and was at its maximum of around 180 min. In another study, by monitoring the stride length of the patients during daily activities, it was reported that the onset of the medication was 24 min. We have obtained almost the same value, as it can be observed in Fig. 8, there was an increase in the number of Exceptional Strides 0.44 h or 26 min after the medication intake. As expected, Exceptional Strides occurred more frequently in ON state periods compared to the OFF state periods (Table 2). Our initial assumption of ON state periods in which we considered between 1 and 3 h after taking the medication was generalized to the whole population. However, such a generalization might not be accurate for an individual patient due to different treatment responses. Moreover, the emergence of Exceptional Strides in less than half an hour for some patients (Fig. 8) might suggest that the initial assumptions for OFF state periods might not be true. Therefore, having the information about patients’ performance during daily activities and comparing it to their capacity in the clinic can provide the potential to determine and monitor the effect of Levodopa in PD patients in a personalized manner. This is again in favour of the complementary aspect of information from daily living measurements.

It was observed that the occurrence of the Exceptional Strides was hardly seen in short walking bouts as only <1% of them happened during this type of walking bout. This percentage was increased in medium and long walking bouts with long walking bouts having a large portion of the Exceptional Strides (almost 90%). This can be justified by the fact that shorter walking bouts might occur when there are obstacles in the walking path of the individuals making them pause or stop their gait. Furthermore, shorter walking bouts can occur when people are doing several daily tasks requiring more attention and as a consequence causing the reduction of gait speed. However, for longer walking bouts, people can reach a more steady-state gait speed where it can be expected that the main task of walking is less perturbed by secondary tasks as is the case in the clinical assessment^28^. The importance of considering longer walking bouts to predict PD has also been shown in another study^34^. It was shown that short walking bouts of <20 s cannot reveal a significant difference between the control group and PD patients’ gait speed. However, as the duration of the walking bouts increases, the corresponding gait speed difference between the control and PD group becomes larger, reaching its maximum for walking bouts of longer than 2 min.

Finally, we observed that the Exceptional Strides’ gait speed deviated between −0.06 and 0.04 (Table 2). Therefore, the threshold of 0.1 m/s to consider Exceptional Strides seems reasonable as it lay outside these two values. This threshold was considered only due to the error of our gait speed estimation system. However, to take into account also the performance of the patients individually, an adaptive threshold based on each patient’s gait speed range can be employed.

The main contribution of the current study was a new approach to compare clinical and home assessments, firstly, by comparing the bimodal distribution of gait speed between clinic and home, and secondly, by the Exceptional Strides. These approaches could preserve the information regarding the type of walking bouts, the medication effects, the time of the day as well as the complex distribution of gait speed that has been mostly limited in the literature to a unimodal distribution. Thanks to these two approaches, we were able to determine the conditions that lead patients to reach their capacity. In this way, the clinicians can know to what extent the patients’ capacity is being used during daily activities, especially if a walking test in the clinic is not possible and patients are being monitored remotely in their domestic environment due to situations such as the COVID-19 pandemic^48^. Looking specifically at the difference between the higher preferred gait speed at home and clinic (${{\Delta }}_{\mu _2}$), the 97th percentile of gait speed distribution at home (because we showed Exceptional Strides compose 3% of the gait cycles), walking bouts longer than 1 min, gait cycles happening in the morning, and gait cycles around 3 h after taking the medication has the potential to give some information about the capacity of the patients.

Moreover, to the best of our knowledge, there is no previous work investigating the effect of medication on the difference between clinical and home assessments of gait speed. The comparison of bimodal gait speed distribution between the clinic and the home was shown to have the potential to estimate the optimal number of medication doses throughout the day. Moreover, the effect of medication intake can be monitored objectively by comparing capacity and performance. This can help the clinicians to design the optimal dose of the medication for the patients. Yet more evidence in a larger dataset including healthy controls is needed to determine a meaningful relationship between the number of Exceptional Strides and the stage of PD.

The first limitation of our study was that daily activity assessments have been performed only in one day. Several days or a week could be more relevant to capture all the aspects of daily activities as people may have different amounts of activity, e.g., on weekdays and weekends^38^.

Another limitation of this study was neglecting very short walking bouts, i.e., walking bouts having <15 s duration as these very short walking bouts compose most of the walking bouts during daily activities^34^. These walking bouts could have improved probably the power of calculations. Nevertheless, removing those very short walking bouts made our analysis fairer and also let us obtain a more steady-state gait speed during home assessment.

We did not distinguish curved walking bouts from straight walking bouts during daily activities. An algorithm such as the one introduced by reference^49^ can be employed to detect turnings during daily activities and differentiate the walking bouts during curved and straight paths. As this algorithm was designed for an IMU on the lower back, a sensor on the lower back can be useful for this purpose. Furthermore, the effect of the duration of the walking bouts on the comparison between clinic and real-life should also be studied.

Finally, in the current study, we investigated the circumstances in which the clinical and daily living measurements were more associated together. Although the findings can help the clinicians to know which tests in the clinic are better representative of daily living measurements, or vice versa, which conditions during daily living are better indicative of capacity in the lab, they do not concern about the information that is not mutual between clinic and home.

To conclude, this study presented new insights to investigate when daily activity performance reaches the functional capacity as measured in the clinic. By collecting all walking bouts and estimating their speed, we found that PD patients had a bimodal gait speed distribution during real-life conditions with on average similar modes as the gait tests performed in clinic during various conditions and speeds. Further analysis at stride level showed a low percentage of strides (~3%) had a gait speed equal or greater than the maximum speed in clinic considered as patients’ capacity. These strides, termed as Exceptional Strides, happened mostly before noon, during ON state, and walking bouts with at least 1-min duration. There was an increase in the number of Exceptional Strides starting 26 min after medication intake reaching the maximum at 3 h. It was also concluded that by comparing the capacity and performance, one can monitor the effect of medication during daily activities and possibly adapt it to reach a gait speed closer to that of the capacity more frequently. Future research is however necessary to determine the meaningful relationship between the number of Exceptional Strides and the progression of the disease as well as the amount of Levodopa intake.

## Methods

### Participants and study design

A total of 27 participants (11 females, 16 males) diagnosed with PD based on the UK Brain Bank criteria^50^ were included in the study. Measurements were taken from distinct individuals. Information about demographic data and patients’ characteristics was collected from the participants (age: 70 ± 7.7 years, H&Y stage median of 2, disease duration of 7 ± 5 years, the age of disease onset: 63 ± 8.2). UPDRS including the subscales of UPDRS-II and III was obtained during both ON and OFF medication states by a clinician that was not blinded to the medication status of the patients (UPDRS II of 5.6 ± 4.5 during ON medication and 8 ± 5.9 during OFF medication, UPDRS III of 14.3 ± 10 during ON medication and 25 ± 11.8 during OFF medication). The exclusion criteria were being older than 90 years, suffering from dementia or mobility-related health problems other than PD, the inability to walk consecutively for 20 m, and having a difference of <2 between ON and OFF states in the UPDRS-III to take into account minimal clinically significant difference^51^. The study was approved by the institutional review board of Centro Hospitalar Universitário do Porto (Porto, Portugal) and was performed in agreement with the WMA Declaration of Helsinki’s Ethical Principles for Medical Research Involving Human Subjects^52^. Written informed consent was collected from all the patients before their participation.

### Clinical assessments

Patients were evaluated first at OFF state which occurred at least 12 h after their last medication intake. The patients were equipped with RehaGait (Hasomed GmbH, DE) with IMUs on each foot. After at least one hour from their medication intake, patients were considered to be in their ON medication state and were evaluated again. During each medication state, they were asked to perform a 20-m straight walk test at a convenient and fast speed as well as circular walking tests (1080° around a circle) at both left and right directions. However, due to the difficulties of the patients to complete the straight walking test at fast speed, this test was skipped during OFF. The clinical gait assessments are summarized in Table 3.

**Table 3:** Clinical gait tests.

ON state	OFF state
20-m straight walking test at convenient speed	20-m straight walking test at convenient speed
20-m straight walking test at fast speed	
Circular walking test at left direction	Circular walking test at left direction
Circular walking test at right direction	Circular walking test at right direction

### Home assessment

The next day, patients came to the hospital again around 9:00 in the morning to be equipped with Physilog® 5 (Gait Up, CH) IMUs on the right foot. The patients were asked to go back home and perform their daily routine activities for one day. It should be noted that patients were allowed to go outside the home and perform their usual daily activities. Therefore, “home assessment” can also include daily activities that had been done outside their living space. The sensors were programmed to start recording automatically at 10:00 for 12 h, i.e., until 22:00. The patients recorded the time of their medication intake in a diary. Based on their diary, we have assumed and defined the ON state periods as starting one hour after taking the medication and lasting for a period of two hours and the OFF state periods starting half an hour before taking the medication and lasting for a period of one hour^53,54^.

### Gait speed and walking bout extraction

For all of the clinical gait tests mentioned in Table 3, the raw data of gyroscope and accelerometer from both of the feet were used. To have a more steady-state gait, the first and last two strides were discarded. With a previously validated algorithm^45^, gait speed was obtained for each gait cycle by the right foot IMU. Since each of the clinical tests (Table 3) contained only one walking bout, no analysis regarding the detection of walking bouts was made as opposed to the home assessment. In addition to the gait speed for each gait cycle, the mean value of the gait speed throughout the test was also calculated.

For home assessments, first, the walking bouts were detected using the angular velocity signal^55^. To have enough steps within each walking bout, the walking bouts that had a duration of <15 s were discarded. This was done to prevent detecting other movements than gait that can impact our analysis wrongly. Furthermore, removing very short walking bouts let us have a more steady-state gait during daily activities. Next, within each walking bout, gait speed was calculated for each gait cycle^45^. Gait cycles with a speed of <0.2 m/s were discarded as these could potentially be a break.

Walking bouts were divided into short (duration between 15 and 30 s), medium (duration between 30 and 60 s), and long (duration of more than 60 s) bouts.

### Distribution of gait speed at the clinic and home

To obtain a distribution for the gait speed, all the gait cycles were considered for each clinical and home setting. There is some evidence in the literature for a bimodal Gaussian distribution during daily-life gait speed^31^ and cadence^42^. As in the current study we had performed several clinical gait tests in various conditions, we considered the following bimodal distribution $f(x)$ for each of the clinical and home assessments.

1 $$f\left( x \right) = c_1{\mathrm{exp}}\left( { - \frac{1}{2}\left( {\frac{{x - \mu _1}}{{\sigma _1}}} \right)^2} \right) + c_2{\mathrm{exp}}\left( { - \frac{1}{2}\left( {\frac{{x - \mu _2}}{{\sigma _2}}} \right)^2} \right)$$

in which *x* is the gait speed distribution, $c_1$ and $c_2$ determine the amplitude, $\mu _1$ and $\mu _2$ are the means presenting the preferred lower and higher gait speed^31^, and $\sigma _1$ and $\sigma _2$ are the standard deviations from each of the means.

MATLAB’s fitgmdist function was used to fit the Gaussian models. Ashman’s D was calculated to quantify the fitting quality. A value of >2 is indicative of a bimodal distribution^56^.

The two means and standard deviations were compared together between clinical and home assessments using a two-sided *t*-test for normally distributed data or Wilcoxon rank sum test for data that did not follow a normal distribution. One-sample Kolmogorov–Smirnov test was used to test for the normality of data. Pearson’s correlation coefficient with the criteria given in^57^ for low, moderate, and high correlations was also obtained.

To observe the differences between the preferred gait speeds at clinic ($\mu _{1,{\mathrm{clinic}}}$, $\mu _{2,\mathrm{clinic}}$) and at home ($\mu _{1,\mathrm{home}}$, $\mu _{2,\mathrm{home}}$), we defined two parameters ${{\Delta }}_{\mu _1}$ and ${{\Delta }}_{\mu _2}$ that represnet the percentage of difference between clinic and home for $\mu _1$ and $\mu _2$, respectively.

2 $$\Delta _{\mu _1} = \frac{{2\left( {\mu _{1,\mathrm{clinic}} - \mu _{1,{\it{\mathrm{home}}}}} \right)}}{{\mu _{1,\mathrm{clinic}} + \mu _{1,{\it{\mathrm{home}}}}}} \times 100$$

3 $$\Delta _{\mu _2} = \frac{{2\left( {\mu _{2,\mathrm{clinic}} - \mu _{2,{\it{\mathrm{home}}}}} \right)}}{{\mu _{2,\mathrm{clinic}} + \mu _{2,{\it{\mathrm{home}}}}}} \times 100$$

We obtained Pearson’s correlation coefficient between number of doses and ${{\Delta }}_{\mu _1}$ and ${{\Delta }}_{\mu _2}$ considering all the patients.

Furthermore, the cumulative distribution function of gait speed at the clinic (${\mathrm{CDF}}_{\mathrm{clinic}}$) as well as home (${\mathrm{CDF}}_{\mathrm{home}}$) were determined for each patient. Receiver operating characteristic (ROC) curve was obtained for each patient by considering ${\mathrm{CDF}}_{\mathrm{home}}$ as the *x* axis and ${\mathrm{CDF}}_{\mathrm{clinic}}$ as the *y* axis. Finally, for each patient, the area under the ROC curve (AUC) was calculated. An AUC value close to 0.5 means that the clinical and home assessments have the same gait speed distribution while a value closer to 0 (or 1) means that the probability of having a gait speed less than a specific value is higher at home (or in the clinic).

### Capacity vs. performance (Exceptional Strides)

For each patient, their average gait speed during the 20-m walk test with fast speed (at ON medication) was obtained and taken as their capacity ($V_c$). To investigate when patients reach their capacity $V_c$ or go beyond it during daily activities, for each stride $k$, its gait speed ($V_{h,k}$) was compared to $V_c$ and if it was greater or equal than $V_c$, it was marked as an Exceptional Stride and the following information was extracted for that stride:

- Time of occurrence ($t_k$)
- Its time difference compared to the last medication intake ($t_k - t_c$)
- Whether it happened during ON state or OFF state (${\mathrm{MED}}_k$)
- The duration of its corresponding walking bout ($T_{WB,k}$)
- Whether it happened during short, medium, or long walking bout ($WB_k$)
- Its gait speed difference compared to $V_c$ ($V_{h,k} - V_c$)

To correct for measurement errors, a threshold of 0.1 m/s was used when comparing $V_{h,k}$ and $V_c$ to obtain the Exceptional Strides.

The impact of the status of PD on the percentage of Exceptional Strides over the total number of strides for each patient was examined. We calculated the correlation coefficient between the amount of Exceptional Strides and UPDRS-III (at OFF medication) as well as the correlation coefficient between the amount of Exceptional Strides and number of medication intakes during the day.

## Data Availability

The datasets generated during and/or analysed during the current study are not publicly available (no ethical committee approval) but might be available from the corresponding author on reasonable request and the authorization of other co-authors.

## Code availability

The codes written for this study to extract and analyze the parameters are the property of the host institutions under the framework of the Keep Control European Project. They might be available from the corresponding author upon reasonable request and the authorization of other co-authors and host institutions. MATLAB R2020b (MathWorks, US) software was used to extract the parameters and perform the statistical analyses.
